# Transplante Combinado de Coração e Rim Realizado em Etapas com Máquina de Perfusão Pulsátil: Uma Estratégia Viável para o Transplante Combinado

**DOI:** 10.36660/abc.20240007

**Published:** 2024-07-31

**Authors:** Arthur Gus Manfro, Rodrigo Fontanive Franco, Roberto Ceratti Manfro, Nadine Clausell, Livia Adams Goldraich

**Affiliations:** 1 Serviço de Nefrologia Hospital de Clínicas de Porto Alegre RS Brasil Serviço de Nefrologia – Hospital de Clínicas de Porto Alegre, RS – Brasil; 2 Serviço de Cardiologia Hospital de Clínicas de Porto Alegre RS Brasil Serviço de Cardiologia – Hospital de Clínicas de Porto Alegre, RS – Brasil

**Keywords:** Transplante cardíaco, transplante renal, Preservação de Órgãos

## Introdução

Transplantes isolados de coração e de rim tornaram-se procedimentos padrões no tratamento de pacientes com falência de órgão. A disfunção renal é frequentemente observada em pacientes com insuficiência cardíaca (IC) avançada, exercendo um impacto significativo no prognóstico do transplante cardíaco. Em contrapartida, a IC avançada é comum em pacientes com doença renal terminal, o que inviabiliza o transplante renal. Consequentemente, muitos centros têm adotado o transplante renal e cardíaco combinado, como tratamento padrão desses pacientes.^1^ O progresso nessa área levou a um aumento substancial de transplantes de múltiplos órgãos na última década. Apesar do aumento nas realizações dos transplantes combinados, ainda há incertezas quanto à melhor sequência das cirurgias.^2^ No Brasil, enquanto um número significativo de transplantes cardíacos e renais isolados são realizados anualmente, poucos transplantes combinados foram documentados.^3,4^ Considerando os avanços na área, espera-se que os transplantes combinados tornem-se mais comuns em breve.

Apresentamos aqui um relato de caso de um transplante cardíaco e renal combinado, utilizando-se preservação com perfusão pulsátil.

## Relato de Caso

**Figura 1:**
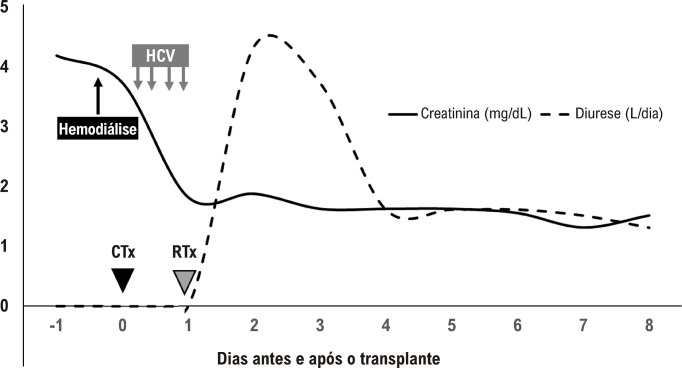
– Procedimentos e evolução da creatinina sérica e débito urinário; HVC: Hemodiafiltração Venovenosa Contínua; CTx: transplante cardíaco; RTx: transplante renal.

Paciente do sexo masculino, 31 anos, com história de meningococcemia em 2014, desenvolveu doença renal em estágio terminal, necessitando de hemodiálise crônica. Concomitantemente, o paciente desenvolveu IC avançada por cardiomiopatia dilatada de etiologia indefinida e disfunção sistólica ventricular esquerda progressiva. Essa condição levou a uma internação hospitalar do paciente em choque cardiogênico (INTERMACS1) em setembro de 2021. Após 45 dias de terapia intensiva envolvendo Hemodiafiltração Venovenosa Contínua (HVC), vasopressores, e antibióticos, o estado do paciente melhorou, possibilitando a transição para suporte inotrópico e hemodiálise intermitente em uma enfermaria equipada com telemetria. Em seguida, as condições do paciente para o transplante foram avaliadas e otimizadas. Duas semanas após sua inclusão na lista de transplante, surgiu um doador compatível disponível. Um transplante cardíaco ortotópico foi realizado com um tempo de *bypass* cardiopulmonar de 90 minutos e tempo de isquemia do enxerto de 260 minutos. Simultaneamente, o rim esquerdo do doador foi colocado em uma máquina de preservação de perfusão renal (Organ Recovery Systems; Itasca, EUA). Após o transplante cardíaco, o paciente recebeu suporte hemodinâmico e HVC. Vinte e duas horas depois, com condições hemodinâmicas adequadas, o enxerto renal foi implantado. Ao final do procedimento, com 29 horas de isquemia total do rim, o paciente apresentou diurese espontânea, e não houve necessidade de diálise no pós-operatório (Figura 1). A estratégia de imunossupressão usada foi indução com timoglobulina, e manutenção com tacrolimo, micofenolato, e prednisona. O paciente recebeu alta do hospital após 21 dias. O paciente permanece sem sintomas cardíacos dois anos após o transplante e é acompanhado regularmente no ambulatório de transplante. Apresenta creatinina sérica de 1,1 mg/dL, sem distúrbios eletrolíticos ou proteinúria. O ecocardiograma mostra fração de ejeção de 59%. O paciente não desenvolveu nenhum episódio de rejeição cardíaca ou anticorpos específicos do doador.

## Discussão

O transplante cardíaco e renal combinado é um procedimento complexo, com o potencial de melhorar significativamente as taxas de sobrevida e a qualidade de vida de pacientes selecionados. Identificar quais pacientes se beneficiariam dessa abordagem ou de um transplante sequencial continua sendo um tema de pesquisa.^5,6^

Nos últimos anos, vários estudos investigaram estratégias para determinar o momento ótimo para o transplante renal em pacientes com doença renal crônica (DRC). Tais investigações demonstraram que o transplante combinado é justificável para pacientes com DRC avançada – taxa de filtração glomerular estimada (TFGe) < 30mL/min – que requerem um transplante cardíaco considerando o risco substancial de progressão da DRC após o transplante cardíaco. Um extenso relatório da *United Network Sharing* (UNOS) revelou que uma TFG < 37 mL/min foi associada a desfechos favoráveis em receptores de transplantes combinados em comparação a receptores de transplante cardíaco isolado.^7^ No entanto, é crucial reconhecer que o procedimento combinado tem um risco elevado e, por isso, os potenciais benefícios devem ser ponderados com cuidado. Além disso, do ponto de vista ético e de saúde pública, a alocação de dois órgãos simultâneos para um único receptor deve ser bem avaliada, particularmente no contexto de escassez de órgãos.^8^

Ao optar por uma estratégia de transplante combinado, deve-se planejar com cuidado a cirurgia, pois existem diferenças notáveis entre um procedimento simultâneo e um procedimento em etapas. No primeiro, ambos os órgãos são implantados em um mesmo procedimento cirúrgico, enquanto no segundo, o rim é preservado em uma solução, preferencialmente em um aparelho de perfusão pulsátil. Em uma abordagem simultânea, o tempo de isquemia do enxerto renal é minimizado, e o rim é geralmente implantado em condições clínicas menos favoráveis. Por outro lado, uma estratégia em etapas permite um tempo adequado para se alcançar estabilidade hemodinâmica e, ao mesmo tempo, assegurar que o rim seja apropriadamente preservado *ex-vivo*. Em comparação a uma estratégia sequencial tradicional, esta abordagem provavelmente impõe um peso imunológico menor, uma vez que ambos os órgãos são do mesmo doador.^9^Ainda, diferentemente da abordagem combinada, se as condições hemodinâmicas forem inadequadas para o transplante renal, o órgão pode ser realocado na lista de espera. Essas diferenças estão resumidas na Tabela 1.

**Tabela 1:** – Principais diferenças entre alternativas cirúrgicas para o transplante cardíaco e renal a partir de um único doador

	Técnica cirúrgica	
	Simultânea	Em etapas
Status hemodinâmico durante o implante do enxerto renal	Instável	Estável
Tempo de isquemia do enxerto renal	Reduzido	Aumentado
Alocação e uso do órgão do doador	Alocação de ambos os órgãos para um único receptor	Possibilidade de alocação do enxerto renal para um recipiente diferente em caso de desfecho desfavorável do transplante cardíaco

A experiência brasileira em transplante combinado ainda está nos estágios iniciais. Atik et al.^3^ documentaram uma série bem-sucedida de quatro transplantes simultâneos de coração e de rim ao longo de 12 anos. Esses procedimentos foram realizados em pacientes com cardiomiopatia chagásica que já realizavam hemodiálise crônica.^3^ Em nosso conhecimento, o presente caso representa o primeiro procedimento de transplante renal e cardíaco combinado no Brasil, a partir de um mesmo doador, empregando a técnica de preservação pulsátil. Enquanto o Brasil e a América Latina registram suas primeiras experiências em transplantes combinados, esta abordagem cirúrgica tornou-se mais comum na Europa e principalmente nos Estados Unidos. Dados de centros de transplante mostram algumas diferenças dessas estratégias quando comparadas a procedimentos de transplantes de um único órgão. Geralmente, os doadores para procedimentos combinados são mais jovens, o tempo de internação no pós-operatório é mais longo, e as estratégias de imunossupressão mais agressivas. Neste cenário, a incidência de rejeição aguda é mais baixa, e as principais causas de mortalidade do receptor são complicações infecciosas. Ainda faltam comparações diretas entre essas estratégias de transplante em diferentes regiões do mundo, considerando a baixa proporção desses transplantes em países de renda baixa e média.^10,11^

Em conclusão, nosso objetivo foi ilustrar a viabilidade de uma abordagem em etapas de um transplante cardíaco e renal combinado. Essa abordagem mostrou-se viável com um planejamento cuidadoso, enfatizando a otimização das condições clínicas do receptor, e a disponibilidade de uma máquina de perfusão de órgão, permitindo um tempo adequado para estabilização hemodinâmica entre os procedimentos de transplante. Os centros devem contribuir ativamente relatando dados pertinentes para melhorar o entendimento e o refinamento desta abordagem inovadora de transplante.
