# Supplementary material for: Transplante Combinado de Coração e Rim Realizado em Etapas com Máquina de Perfusão Pulsátil: Uma Estratégia Viável para o Transplante Combinado
Source: Arq Bras Cardiol. 2024 Jul 31;121(7):e20240007. [Article in Portuguese] doi: 10.36660/abc.20240007 (PMC11464091; doi:10.36660/abc.20240007)
Supplement: Translation

# Translation: Staged Heart-Kidney Transplantation with Renal Pulsatile Preservation: A Viable Approach for Combined Transplantation

**DOI:** 10.36660/abc.20240007i

**Authors:** Arthur Gus Manfro, Rodrigo Fontanive Franco, Roberto Ceratti Manfro, Nadine Clausell, Livia Adams Goldraich

**Affiliations:** 1 Brazil Serviço de Nefrologia – Hospital de Clínicas de Porto Alegre, RS – Brazil; 2 Brazil Serviço de Cardiologia – Hospital de Clínicas de Porto Alegre, RS – Brazil

**Keywords:** Heart Transplantation, Kidney Transplantation, Organ Preservation

## Introduction

Isolated heart and kidney transplants have become routine procedures for addressing end-stage organ failure. Notably, renal dysfunction is frequently observed in patients with advanced heart failure (HF), significantly impacting the prognosis of heart transplantation. Conversely, advanced HF is common in patients with end-stage renal disease (ESRD), often impairing kidney transplantation. Consequently, many centers are adopting combined heart and kidney transplantation (HKTx) as the standard of care for those patients.^1^ The progress in this field has led to a substantial increase in multiorgan transplants over the last decade. Despite the growth in HKTx, uncertainties persist regarding the preferred surgical sequence.^2^ In Brazil, while a significant number of isolated heart and kidney transplants are performed annually, only a few combined HKTx have been documented.^3,4^ Considering the advancements in the field, it is anticipated that combined transplants will become more commonplace shortly. Here, we present a case report of a staged HKTx with renal pulsatile perfusion preservation.

## Case Report

A 31-year-old male patient, with a history of meningococcemia in 2014, developed ESRD requiring chronic hemodialysis. Concurrently, he developed advanced HF due to dilated cardiomyopathy of undefined etiology and progressive left ventricular systolic dysfunction. This condition led to a hospitalization in September 2021 with INTERMACS 1 cardiogenic shock. Following 45 days of intensive care involving continuous veno-venous hemodiafiltration (CVVHDF), vasopressors, and antibiotics, the patient’s status improved, allowing for transition to inotropic support and intermittent hemodialysis in a telemetry-equipped ward. Subsequently, the patient’s conditions for transplantation were assessed and optimized. Two weeks after his inclusion on the transplant list, a compatible donor became available. An orthotopic heart transplantation was performed with cardiopulmonary bypass time of 90 minutes and graft ischemic time of 260 minutes. Simultaneously, the donor’s left kidney was placed in a pulsatile renal perfusion and preservation machine (Organ Recovery Systems; Itasca, USA). Following the heart transplant, the patient received hemodynamic support and CVVHDF. After 22 hours, with optimized hemodynamic conditions, the renal graft was implanted. At the end of the procedure, with 29 hours of total kidney ischemia, the patient presented with spontaneous diuresis, obviating the need for postoperative dialysis (Figure 1). The immunosuppression strategy employed was thymoglobulin induction, and maintenance with tacrolimus, mycophenolate, and prednisone. The patient was discharged from the hospital within 21 days. He remains free of cardiac symptoms at two years post-transplant and undergoes regular follow-ups in the transplant outpatient services. His serum creatinine is 1.1 mg/dL, without electrolyte disorders or proteinuria. Echocardiography shows an ejection fraction of 59%. He has not developed any cardiac rejection episodes or donor specific antibodies.

**Figure 1:** – Procedures and evolution of serum creatinine and urinary output; CVVHDF: continuous venovenous hemodiafiltration; HTx: heart transplantation; KTx: kidney transplantation.

## Discussion

Combined HKTx is a complex procedure with the potential to significantly improve survival rates and the quality of life in selected patients. The crucial task of identifying which patients would benefit from this approach or from a sequential transplantation remains a subject of active research.^5,6^

In recent years, numerous studies have investigated strategies to determine the optimal timing for kidney transplants in heart recipients with chronic kidney disease (CKD). These investigations have demonstrated that HKTx may be justifiable for patients with advanced CKD (estimated glomerular filtration rate, eGFR <30mL/min) requiring a heart transplant considering the substantial risk of CKD progression post-heart transplant.^6^ An extensive report from the United Network Organ Sharing (UNOS) revealed that a GFR <37 mL/min was associated with favorable outcomes in HKTx recipients compared to those undergoing isolated heart transplants.^7^ However, it is crucial to acknowledge that the combined procedure carries an elevated risk, and therefore, the potential benefits should be carefully weighed. Additionally, from a public health and ethical standpoint, the allocation of two simultaneous organs for a single recipient must be thoroughly evaluated, particularly in the context of organ shortage.^8^

When opting for a combined HKTx strategy, careful consideration must be given to the planned surgery, as there are notable distinctions between a simultaneous and a staged procedure. In the former, both organs are implanted in a single surgical procedure, while in the latter, the kidney is preserved in a solution, preferably within a pulsatile perfusion device. In a simultaneous approach, ischemic time of the renal graft is minimized, and the kidney is often implanted under less favorable clinical conditions. On the other hand, a staged strategy allows for adequate time to achieve hemodynamic stability, concurrently ensuring the kidney is appropriately preserved *ex-vivo*. Compared to a traditional sequential strategy, this approach likely imposes a lesser immunologic burden, given that both organs are from the same donor.^9^ Additionally, unlike the combined approach, if hemodynamic conditions are inadequate for the kidney transplant, the organ can be reallocated to the waiting list. These distinctions are summarized in Table 1.

**Table 1:** – Main differences between surgical alternatives for single-donor combined heart and kidney transplantation

	Surgical technique	
	Simultaneous	Staged
Usual hemodynamic status during renal graft implant	Unstable	Stable
Renal graft ischemic time	Reduced	Increased
Allocation and donor organ utilization	Allocation of both organs to a single recipient	Possibility of kidney graft allocation to a different recipient in case of unfavorable heart transplant outcome

The Brazilian experience in HKTx is still in its early stages. Atik et al.^3^ documented a successful series of four simultaneous heart-kidney transplants conducted over 12 years. These procedures were carried out in patients with Chagas’ cardiomyopathy who were already undergoing chronic hemodialysis.^3^ To the best of our knowledge, the present case represents the first staged HKTx procedure performed in Brazil from a single donor, employing the renal pulsatile perfusion preservation technique. While Brazil and Latin America are documenting their first experiences in combined transplants, this surgical approach has become more common in Europe and mainly in the United States. Data from transplantation centers show some differences from these strategies when compared to single organ transplant procedures. Donors for combined procedures are usually younger, post-operative stay is longer, and immunosuppression strategies are usually more aggressive. In this scenario, incidence of acute rejection is lower, and the main causes of recipient mortality are infectious complications. Direct comparisons between such transplantation strategies in distinct world regions are still lacking, considering the low proportion of such transplants in low-to-middle income countries.^10,11^

In conclusion, we aimed to illustrate the feasibility of a staged approach for a combined HKTx. This approach proves viable with a careful planning, emphasizing the optimization of recipient clinical conditions, and the availability of an organ perfusion machine, allowing adequate time for hemodynamic stabilization between the transplant procedures. Centers should actively contribute by reporting pertinent data to enhance the understanding and refinement of this innovative transplantation approach.
